# Lower rate of delayed graft function is observed when epidural analgesia for living donor nephrectomy is administered

**DOI:** 10.1186/s12871-019-0713-y

**Published:** 2019-03-18

**Authors:** Wolfgang Baar, Ulrich Goebel, Hartmut Buerkle, Bernd Jaenigen, Kai Kaufmann, Sebastian Heinrich

**Affiliations:** 1 Department of Anesthesiology and Critical Care, Medical Center, Faculty of Medicine, University of Freiburg, Hugstetter Strasse 55, 79106 Freiburg, Germany; 2 Department of General and Visceral Surgery, Medical Center, Faculty of Medicine, University of Freiburg, Hugstetter Strasse 55, 79106 Freiburg, Germany

**Keywords:** Kidney transplantation, Delayed graft function, Epidural analgesia, Donor nephrectomy

## Abstract

**Background:**

The beneficial effects of epidural analgesia (EDA) in terms of pain control and postoperative convalescence are widely known and led to a frequent use for patients who underwent living donor kidney nephrectomy. The objective of this study was to determine whether general anesthesia (GA) plus EDA compared to GA only, administered for living donor nephrectomy has effects on postoperative graft function in recipients.

**Methods:**

In this monocentric, retrospective cohort analysis we analyzed the closed files of all consecutive donor- recipient pairs who underwent living donor kidney transplantations from 2008 to 2017. The outcome variable was delayed graft function (DGF), defined as at least one hemodialysis within seven days postoperatively, once hyperacute rejection, vascular or urinary tract complications were ruled out. Statistical analyses of continuous variables were calculated using the two-tail Student’s t test and Fisher exact test for categorical variables with a significance level of *p* < 0.05, respectively.

**Results:**

The study enclosed 291 consecutive living donor kidney transplantations. 99 kidney donors received epidural analgesia whereas 192 had no epidural analgesia. The groups showed balanced pretransplantational characteristics and comparable donors´ and recipients’ risk factors. 9 out of all 291 recipients needed renal replacement therapy (RRT) during the first 7 days due to delayed graft function; none of these donors received EDA. The observed rate of DGF in recipients whose kidney donors received epidural analgesia was significantly lower (0% vs. 4.6%; *p* = 0.031).

**Conclusions:**

In our cohort we observed a significantly lower rate of DGF when epidural analgesia for donor nephrectomy was administered. Due to restrictions of the study design this observation needs further confirmation by prospective studies.

## Background

Living kidney transplantation showed superior results compared to deceased donor kidney transplantation in terms of graft survival, accessibility, waiting time and cost containment for public health services [1–3]. For patients undergoing surgical procedures for another one’s benefit, it is important to minimize perioperative risks and inconvenience. Furthermore, it is the healthcare providers’ duty to maximize the beneficial impact of the donation for the recipient.

In numerous studies major outcome benefits like mortality of EDA could neither be confirmed nor denied [4, 5]. However, the beneficial effects of EDA in terms of intra- and postoperative pain control, intestinal motility, early mobilization and duration of ICU- hospitalization are widely known and find broad acceptance [6–9]. Therefore it is not surprising, that continuous EDA is a mandatory part of many surgical fast track programs [10–12]. In order to provide these advantages also for kidney donors and to increase their convalescence and speed up their reintegration in daily life, we offered EDA to patients for donor nephrectomy, if contraindications were ruled out and patients gave their informed consent. The primary intent of providing perioperative EDA for donor nephrectomy are the beneficial effects for the donor [13–15]. These EDA effects are mostly mediated by perioperative sympathicolysis which probably has effects on the kidney intended for transplantation [16, 17]. Potential effects on graft function of kidneys explanted from donors with EDA in terms of a two day follow up of glomerular filtration rate, microalbuminuria, or creatinine clearance have shown no differences in a small cohort [17]. Potential effects on the incidence of delayed graft function have not yet been reported. Therefore, the aim of this hypothesis generating study was to determine whether GA plus EDA compared to GA only, administered for living donor nephrectomy is associated with beneficial effects on postoperative graft function after transplantation.

## Methods

**Fig. 1:**
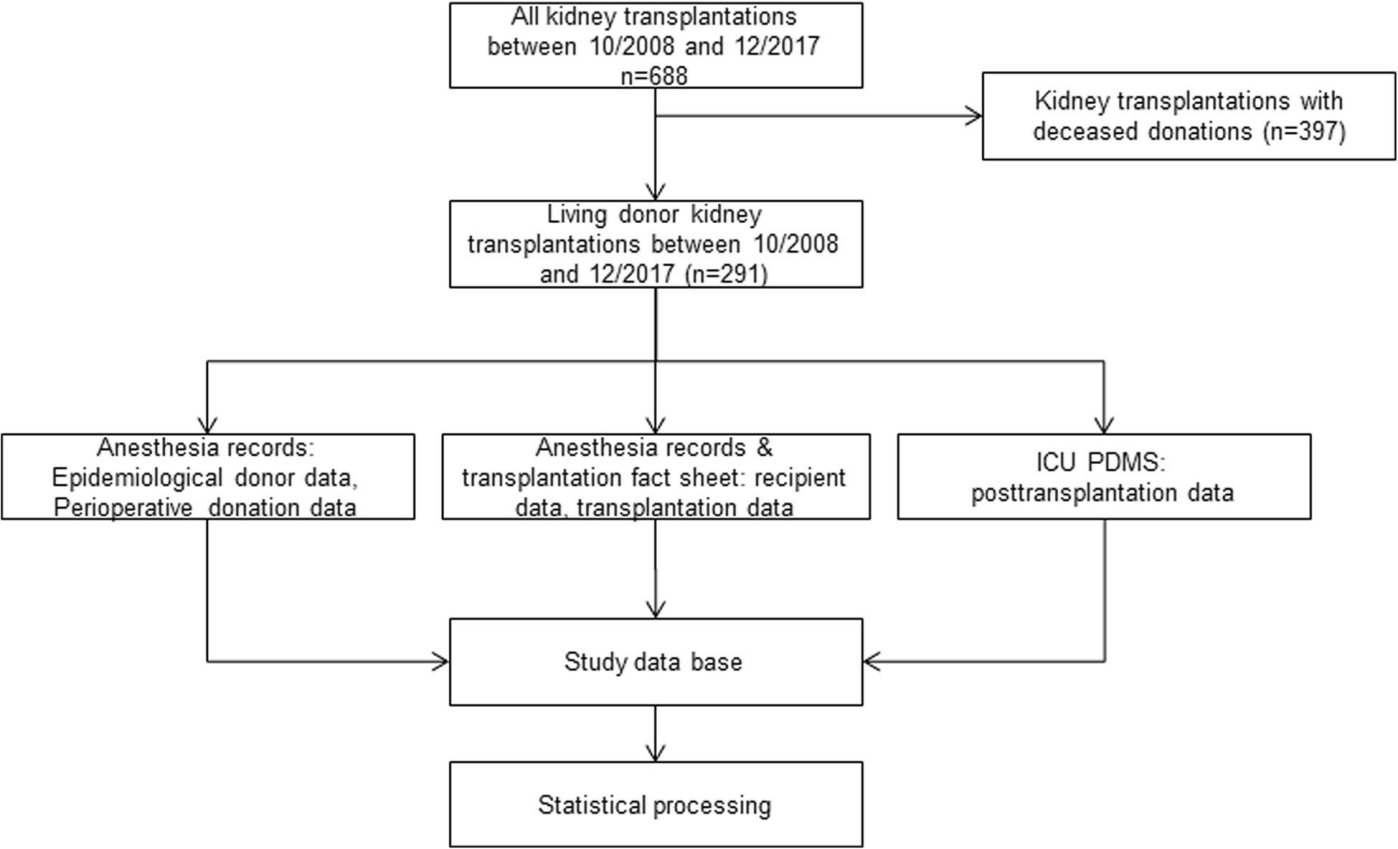
Flowchart showing the data collection of the study

This retrospective cohort study was approved by the local Institutional Review Board, University of Freiburg, Germany (approval number EK 555/17). The study was conducted at the Department of Anesthesiology and Critical Care and the Department of General and Visceral Surgery, Medical Center - University of Freiburg, Faculty of Medicine - University of Freiburg Germany. The study was planned and designed in accordance with the initiative for Strengthening the Reporting of Observational Studies in Epidemiology (STROBE), using the suggested checklist for epidemiological cohort studies [18]. The study was initiated and designed in March 2018; the retrospective data collection was conducted in June 2018. The onset of data collection is analogous to the existence of an electronic patient data management system on ICU which enabled data acquisition. As we enclosed only closed files and the data collection started in June 2018, cases after December 31th 2017 were not enclosed. The study cohort consists of all consecutive living donor kidney transplantations between October 2008 and December 2017 which determines the sample size. A priori sample size calculation is not applicable in this fully retrospective and observational study design. Figure 1 shows the protocol of data collection and statistical processing.

Recipient and donor evaluation were based on a check-up examination which lead to confirmation of donor suitability. Ahead of transplantation all donor- recipient pairs were evaluated by an ethics committee of the District Medical Association Suedbaden, Germany. A positive vote of this ethics committee was mandatory for transplantation.

Surgical procedure was standardized to a maximum as only two different surgeons contributed to the transplantations in this cohort. The donor nephrectomy was performed in supine position over an open anterior extraperitoneal minimal incision laparotomy. Transplantations were performed in the established technique, to the right iliac fossa of the recipient.

Decision on epidural analgesia was based on the patients´ preference. All patients that received epidural analgesia gave their informed consent on that procedure. Epidural analgesia was performed directly preoperatively according a specific departmental standard operating procedure: Epidural catheter was placed between the 7th and the 11th thoracic intervertebral space, followed by an application of 25 μg sufentanil and 10 ml ropivacaine 0.2%. After the initial dose a continuous epidural application of 45 ml ropivacaine 0.2% mixed with 25 μg sufentanil (= ropivacaine 0.18% and sufentanil 0.5 μg/ml) with an infusion rate of 8 ml/h during the surgery was established. Anesthesia procedures for donor nephrectomy with and without epidural analgesia followed a unique mandatory standard operating procedure (SOP) which differed only regarding the administration of EDA and was performed by a specialized transplantation team. Our transplantation team consists of 8 to 10 attending anesthesiologists, who underwent special training (transplant fellowship) and are used to perform anesthesiology for kidney transplantation in accordance to our mandatory in-house standard operating procedure. Over the years the attending anesthesiologists in charge changed, so that in total a number of 25 anesthesiologists took care of the patients. Postoperatively all donors were transferred to a transplantation ICU. Patients who received epidural analgesia were visited daily by the acute pain service of our department. Epidural catheters were removed between the second and the fifth postoperative day by the acute pain service.

Anesthesia for transplantation was performed without epidural analgesia for the recipient and followed a departmental SOP which was established and revised where necessary in close collaboration between the responsible surgeons and anesthetists The SOP addresses the need for 250 mg prednisolone and 10 g mannitol ahead of reperfusion. With the onset of reperfusion of the transplant kidney 125 mg furosemide were administered. Intraoperative fluid and catecholamine management was performed by the attending anesthetist referring to the SOP.

Renal replacement therapy (RRT) was initiated when patients were threatened by volume overload or increased serum potassium levels. Delayed graft function was defined as any renal replacement therapy in the first postoperative week, once hyperacute rejection, vascular or urinary tract complications were ruled out [19–21] .

### Statistics

The data was collected in a MS Excel™ (Microsoft, Redmond, USA) datasheet. Further statistical processing was performed using SPSS™ (IBM, Armonk, USA). Statistical analyses of continuous variables were calculated using the two-tail Student’s t test and Fisher Exact test for categorical variables with a significance level of *p* < 0.05, respectively.

## Results

The study enclosed 291 consecutive living donor kidney transplantations between October 2008 and December 2017. 99 kidney donors received epidural analgesia whereas 192 had no epidural analgesia. Table 1 shows the distribution of patients, the rate of EDA and the incidence of RRT and DGF for every year. For none of the years RRT or DGF rate are significantly increased. All recipients underwent kidney transplantation due to end stage renal disease. No mortality was reported in either group. In the EDA group no epidural catheter associated complications were found.

**Table 1:** Distribution of patients, rate of EDA, incidence of RRT and DGF throughout the observation period

Year	Number of patients	With EDA (n/%)	RRT within 7 days (n/%)	DGF (n/%)
2008	7	0/0%	0/0%	0/0%
2009	31	1/3%	0/0%	0/0%
2010	33	0/0%	1/3%	1/3%
2011	34	6/18%	3/9%	3/9%
2012	35	15/43%	3/9%	1/3%
2013	32	14/44%	0/0%	0/0%
2014	35	8/23%	2/6%	1/3%
2015	31	14/45%	2/6%	2/6%
2016	30	25/83%	2/7%	1/3%
2017	23	16/70%	1/4%	0/0%

**Fig. 2:**
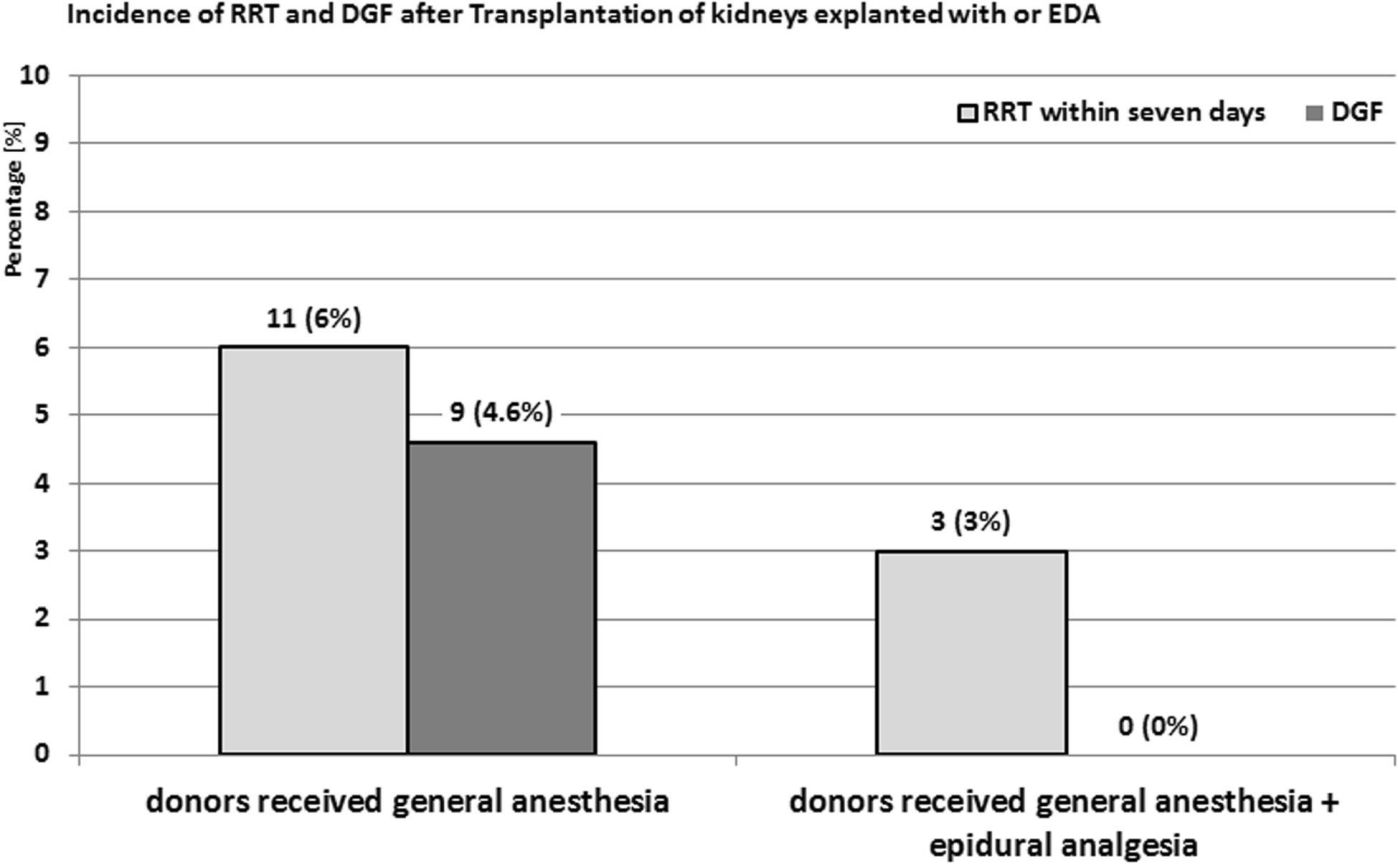
The incidence of renal replacement therapy (RRT) with the first seven days and delayed graft function (DGF) depending on the anesthesia procedure for donor nephrectomy

The perioperative characteristics are shown in Table 2. The two study groups showed no significant differences in several donors´ risk factors except a significantly shorter nephrectomy time (135 vs. 144 min, *p* < 0.003). The intraoperative fluid consumption (1813 vs. 2191 ml; *p* = 0.053) and maximum dose of vasopressor (0.03 vs. 0.06 μg/kg/min; *p* = 0.300) showed no statistically significant difference. None of the recipients´ pre- and intra-transplantation data showed a significant difference (Table 2). After transplantation, 14 out of all 291 recipients needed renal replacement therapy during the first 7 days after transplantation, but only 9 cases due to delayed graft function. The other 5 recipients suffered humoral rejection, thrombosis of the iliac vessel or bleeding complications with the need of a surgical revision (Table 3). All kidney donors to these 9 recipients received GA without epidural analgesia. The incidence of DGF was significantly higher in recipients whose donors did not receive epidural analgesia (4.6% vs. 0%; *p* = 0.031) (Fig. 2). In line with this finding the serum creatinine level as well as the maximum serum potassium level within 7 days were significantly lower in the recipients whose donors received EDA (2.17 vs. 2.04 mg/dl, *p* = 0.036; 5.15 vs. 5.11 mmol/l, *p* < 0.001).

**Table 2:** Main results of the study. Continuous variables are given as mean ± standard deviation, categorical variables are given as absolute number and percentage

	Without EDA (*n* = 192)	With EDA (*n* = 99)	Significance
Donor and nephrectomy data			
Donor male sex [n/(%)]	71 (37%)	32 (32%)	0.469
Donor BMI [kg/m^2^]	25.7 ± 4.1	25.5 ± 3.5	0.165
Donor age [years]	52	52	0.416
Donor preoperative hemoglobin [g/dl]	14.1 ± 1.2	14.1 ± 1.2	0.919
Crystalloid fluid for nephrectomy [ml]	1813 ± 907	2191 ± 1113	0.053
Max. dose of noradrenaline after cut [μg/kg/min]	0.03 ± 0.04	0.06 ± 0.05	0.300
Nephrectomy time (cut – suture) [min]	135 ± 38	144 ± 48	**0.003**
Recipient and transplantation data			
Recipient BMI [kg/m^2^]	24.8 ± 3.9	25.6 ± 4.2	0.168
Recipient male sex [n/(%)]	121 (62%)	61 (62%)	0.899
Recipient age [years]	44 ± 13	47 ± 13	0.853
Recipient rest diuresis [ml]	1098 ± 907	1134 ± 858	0.062
Recipient preoperative creatinine [mg/dl]	8.0 ± 2.8	7.6 ± 2.8	0.744
Duration of transplantation [min]	161 ± 55	145 ± 41	0.129
Warm ischemic period [min]	29 ± 9	26 ± 7	0.138
MAP for anastomosis [mmHg]	93 ± 11	88 ± 16	0.165
Fluid intake during transplantation [ml]	2782 ± 1366	3477 ± 1233	0.559
Posttransplantation data			
Diuresis first hour [ml]	425 ± 430	383 ± 390	0.358
Diuresis 24 h [ml]	9947 ± 5313	10,871 ± 6419	0.062
Recipient creatinine 12–24 h postoperative [mg/dl]	4.37 ± 2.2	4.09 ± 1.8	0.189
Recipient creatinine 36–48 h postoperative[mg/dl]	3.15 ± 2.1	3.04 ± 1.7	0.404
Recipient creatinine 7 days postoperative [mg/dl]	2.17 ± 1.6	2.04 ± 1.1	**0.036**
Max. recipient serum potassium level within 7d	5.15 ± 0.6	5.11 ± 0.4	**0.001**
Renal replacement therapy first postoperative week [n/(%)]	11 (6%)	3 (3%)	0.312
Delayed graft function [n/(%)]	9 (4.6%)	0 (0%)	**0.031**

statistical significance is indicated by bold numbers

**Table 3:** Underlying reasons for renal replacement therapy (RRT) and met definition of delayed graft function (DGF)

Case Number	Year	EDA	Underlying reason leading to RRT within 7 days	DGF
507	2010	no	graft perfusion deficit	yes
581	2011	no	insufficient graft function, later sepsis	yes
588	2011	no	critical potassium levels, good graft function later on	yes
624	2011	no	acute tubules necrosis	yes
666	2012	yes	humoral rejection	no
692	2012	no	bleeding complication, needed operative revision	no
701	2012	no	insufficient graft function	yes
829	2014	no	humoral rejection	no
859	2014	no	graft perfusion deficit	yes
888	2015	no	insufficient graft function, critical potassium levels	yes
935	2015	no	insufficient graft function	yes
1017	2016	no	insufficient graft function	yes
1023	2016	yes	humoral rejection	no
1089	2017	yes	thrombosis of recipients iliac vessel	no

## Discussion

The benefits of EDA regarding pain control, ICU stay, intestinal motility and early mobilization are frequently reported [22–27]. The beneficial effects of EDA in terms of pain control and return to normal daily activities specifically for kidney donors have also been reported in the past [28, 29]. This retrospective cohort study of 291 living donor kidney transplantations compared 99 cases whose donors received EDA with 192 cases that received GA only, with regard to DGF in the recipients. The main result is that DGF is significantly more frequent in patients whose kidney donors did not receive EDA. The incidence of DGF in cohorts of living kidney transplantations varies from 4 to 10% and increases morbidity, healthcare costs, hospitalization times and complicates post- transplantation care [30–33]. DGF predisposes for chronic rejection, chronic allograft nephropathy and seems to be causal for increased rates of graft failure and mortality [34, 35].

In line with the significantly lower rate of DGF in EDA group, we found that serum creatinine level, as well as the maximum serum potassium level within 7 days, were significantly lower in the EDA group. Although these findings are statistically significant, their measured levels and differences in numbers are clinically not of relevance. Even when looking at the decline of the serum creatinine levels over the first two days postoperatively no significant or clinically relevant difference can be found. The recipients of the no-EDA group start at a slightly higher level of serum creatinine which should be taken into account. Further baseline characteristics of donors and recipients showed no statistically significant difference or clinically relevant imbalance between the donors and recipients of both groups. An increased intraoperative fluid and vasopressor consumption in the EDA group could be associated with the EDA mediated inhibition of the sympatho- adrenal response with consecutive vasodilatation. However, neither intraoperative fluid nor vasopressor consumption showed a statistically significant difference in our study.

The standard surgical technique for donor nephrectomy in our institution is an open anterior extraperitoneal minimal incision laparotomy. Open surgical technique for donor nephrectomy is associated with inferior cosmetic result, longer hospitalization and more intra- and postoperative pain with consecutively increased need for pain medication [36, 37]. However, the open surgical approach showed superior results in terms of warm ischemia period, surgical costs, length of operation, intraperitoneal complications, recovery of graft function, recipient anastomosis difficulties and incidence of acute tubulus necrosis [38–40]. It is reported that up to 25% of living kidney donors after open surgical technique nephrectomy suffer from chronification of postoperative pain [41]. A reduction of somatic pain within the first six postoperative weeks is associated with improved mental health of kidney donors [37]. These findings underline the need for EDA from the donors’ perspective. The described clinical benefits of EDA for the donor might lose their relevance and have to be reconsidered in case the surgical approach in our institution changes to laparoscopic technique.

The reasons why kidney grafts fail to function immediately after transplantation when acute rejection, urological or vascular reasons are ruled out are associated with the transplanted kidney. DGF is modulated and caused by complex mechanisms of hypoxic and ischemic injuries and insufficient repair mechanisms [42]. These cascades seem to be induced by the operative trauma and the corresponding physiological stress response during donor nephrectomy. It is known that surgical procedures and the physiological stress response are associated with intra- and postoperative hypercoagulability which results in postoperative thromboembolic and vaso-occlusive events [43, 44]. Increased levels of tissue factor, tissue plasminogen activator, plasminogen activator inhibitor-1, and von Willebrand factor which all contribute to hypercoagulability are reported to be found proximately after surgical stimulus [45]. Due to inhibition of nociceptive and non-nociceptive pathways of sympathetic innervation of the adrenal glands, EDA with local anesthetics leads to a perioperative sympathicolysis [46]. Experimental reports on rats showed a significantly improved microcirculation in the areas of EDA mediated sympathetic blockage [47]. In an ovine model with artificially administered pulmonary embolism therapeutic EDA improved macrohemodynamic parameters [48]. It is also reported that EDA modulates postoperative hypercoagulability by normalizing antithrombin III- activity and a decrease of platelet aggregation [49–51]. We hypothesize that a decreased risk of thrombotic and vasoocclusive events which is mediated by therapeutic EDA could be one of the reasons that we found a significantly increased rate of DGF in the non-EDA group.

The potential benefit of EDA performed for the transplantation could not be investigated by our study group. In our institution the transplantation itself is performed without EDA due to the mandatory immune suppressive therapy and the high incidence of platelet dysfunction in patients suffering from end stage renal disease [52, 53]. Hadimioglu and colleagues found improved clinical results and an attenuated stress response in kidney transplantations performed with EDA and general anesthesia compared to general anesthesia alone [54]. Against the background of these results and the results of our study, we will reconsider our previous approach with regard to the use of EDA in kidney transplants.

### The present study has several distinct limitations

First, the retrospective and non-randomized design implies that a study protocol which addresses randomization on who receives EDA is missing. It is speculative why patients opted for or against EDA, possibly the way whether EDA was offered by the visiting anesthesiologist or rather recommended plays an important role. Perhaps patients who opted for EDA were more trustful of their physicians and therefore had less anxiety or stress levels which may have influenced DGF of their donated kidney. The way EDA was offered to the donors might have been changed throughout the years. We have seen that EDA is more frequently performed in the last years of the program compared to the very early years of the observation period. However, this imbalance of EDA rates throughout the observational period was not associated with an accumulation of RRTs or DGF in the early or the late years of the observation. RRT was initiated by visiting nephrology specialists and the request of the attending ICU physician when patients were threatened by volume overload or increased serum potassium levels. We are fully aware that living donor kidney transplantation is a highly complex procedure. The outcome quality is affected by various confounding variables for which we have not adjusted in our study due to the limited number of cases with DGF.

There are also several slightly different definitions on delayed graft function in literature. In our study DGF was defined as any renal replacement therapy in the first postoperative week, when hyperacute rejection, vascular and urinary tract complications were ruled out. More than 22 different definitions of DGF are described, the most common definition refers to any RRT within the first posttransplantational week [55, 19]. Due to the manageable size of our cohort we could screen every case of RRT for the underlying reasons. Knowing these reasons leading to RRT for every patient, we decided to choose a more specific definition of delayed graft function. Beyond the discussion about the definition we have to state, that in the EDA group none of the patients who received RRT showed graft associated reasons leading to RRT. In no case of the EDA group, graft perfusion deficits or insufficient otherwise unexplainable graft dysfunction led to RRT. Finally, we can report of an association between EDA for donor nephrectomy and a lower rate of DGF in our study. However, we are fully aware that correlation does not proof causality. Especially in a multifactorial context such as living kidney transplantations, larger numbers of prospectively randomized assigned patients are needed to provide stronger evidence.

## Conclusions

In this retrospective cohort study, we found an association between epidural analgesia for living kidney donors and significantly less delayed graft function in the corresponding kidney recipients. These results favor not only the beneficial analgesic effect of epidural analgesia for donors, but also show significant beneficial effects for kidney recipients. As our analysis depends on the authors’ experience, derived from a very low level of evidence with consecutive relevant shortcoming in terms of study design, number of index cases and adjustment of confounding variables, our findings have to be confirmed by prospective randomized trials.

## Abbreviations

DGF: Delayed graft function
EDA: Epidural analgesia
GA: General anesthesia
RRT: Renal replacement therapy
SOP: Standard operating procedure
STROBE: Strengthening the reporting of observational studies in epidemiology
